# Can capabilities be self-reported? A think aloud study

**DOI:** 10.1016/j.socscimed.2013.03.035

**Published:** 2013-06

**Authors:** Hareth Al-Janabi, Thomas Keeley, Paul Mitchell, Joanna Coast

**Affiliations:** a Health Economics Unit, University of Birmingham, UK; b MRC Hub for Trials Methodology Research, University of Birmingham, UK

**Keywords:** Capability approach, EQ-5D, Health economics, ICECAP-A, Outcome measurement, Think-aloud, United Kingdom, Wellbeing

## Abstract

Direct assessment of capability to function may be useful in healthcare settings, but poses many challenges. This paper reports a first investigation of the feasibility of individuals self-reporting their capabilities and the meaning of the responses. The study was conducted in 2010, using think-aloud interviews with participants in the UK. The findings of the study suggest that the majority of participants were able to comprehend questions about their capabilities, felt able to judge their own capability wellbeing and provided responses in line with this judgement. In a number of cases, for example in relation to ‘autonomy’, participants highlighted that their capability was potentially greater than their functioning. The findings also show varying interpretations of the capability concept, with some participants finding the capability concept unintuitive in relation to specific aspects of life (in particular, ‘attachment’). The findings suggest that guiding individuals in the process of identifying their capabilities may be important in generating consistent responses to capability questions.

## Highlights

• Think-aloud interviews were used, in the UK, to study the response to capability wellbeing questions.
• Most people felt able to judge their capabilities and made few obvious ‘errors’ in doing so.
• The capability concept was unintuitive for some people in some aspects of their life.
• Occasional divergence between capability and functioning was reported.

## Introduction

The notion of ‘capabilities’ as a metric for judging wellbeing is closely associated with the work of Amartya Sen (Sen, 1993, 2009). Capabilities are generally taken to represent the ‘real opportunity’ to achieve things in life that a person ‘has reason to value’ (Sen, 2009, p. 231). Nussbaum, another pioneering figure in this area, suggests that capabilities represent ‘what people are actually able to be and do’ (Nussbaum, 2000, p.5). What constitutes an important capability is open to debate, and is potentially dependent on the context (Sen, 2005). Nevertheless some distinction is drawn between basic capabilities, such as an ability to be well nourished and, higher, more complex capabilities, such as an ability to be socially integrated (Sen, 1993).

The focus on assessing what an individual is able to do, rather than what they end up doing, is intended to incorporate the importance of free choice into welfare assessment. The capability approach, pioneered in human development work (Nussbaum, 2000; Sen, 1987, 1999), has more recently been applied to study health policy issues. In the health field, for example, the capability approach has variously been used to examine the right to die (Anand, 2005), conceptualise health and disability (Law & Widdows, 2008; Mitra, 2006; Ruger, 2010) and understand treatment for, and recovery from, illness (Ferrer & Varela Carrasco, 2010; Hopper, 2007). In health economics there has also been recent interest in using the capability approach to measure the effectiveness and cost-effectiveness of healthcare interventions (Coast, Smith, & Lorgelly, 2008; Cookson, 2005; Entwistle, Firnigl, Ryan, Francis, & Kinghorn, 2012; Grewal et al., 2006; Lorgelly, Lawson, Fenwick, & Briggs, 2010).

To date, most efforts to measure capabilities have focused on measuring functionings (what people actually do) as proxies for what people can potentially do (Chiappero-Martinetti & Roche, 2009). Approaches that measure functioning provide valuable information on wellbeing and can often utilise existing datasets, but are clearly hampered by only being proxy measures of true capability. Recently interest has grown in developing tools that ask individuals to self-report their capabilities. Some of these tools seek to elicit individuals' capability in complex areas, such as ‘attachment’ or ‘enjoyment’ in life (Al-Janabi, Flynn, & Coast, 2012; Coast, Flynn, et al., 2008). Others focus on more specific capabilities, for example, Anand et al. (2009) developed over 50 capability indicators from the British Household Panel (now Understanding Society) Survey, which include questions, for example, about individuals' ability to eat certain food and access family planning interventions.

There are some reasons to be sceptical that capabilities can be meaningfully self-reported. Sen himself points out that self-reported data is subject to undesirable adaptation, with a key concern being that individuals with lower expectations of life will under-report problems with their wellbeing (Sen, 2002). In general terms, self-reported quality of life data can be subject to ‘response shift’ (Sprangers & Schwartz, 1999). Response-shift theory suggests that a catalyst, such as a period of poor health, acts on individuals' perceptions of their quality of life in a way that can induce a change in the way that individuals respond to quality of life questions (in addition to any ‘real’ change in their quality of life). Thus observed differences (between and within individuals) in reported quality of life cannot be attributed solely to ‘real’ change. A second concern with self-reported data is that individuals misunderstand terms in questionnaires and provide misleading answers as a result. Often, even with relatively simple terms, individuals' interpretations of the meaning can differ from that intended by the researcher (Schober, Conrad, & Fricker, 2004). Even apparently simple terms like ‘ill’ and ‘healthy’ can mean different things to different people (Donovan, Frankel, & Eyles, 1993; Mallinson, 2002).

Both these adaptation and comprehension issues are potential problems, whether the aim is to assess capabilities or other conceptualisations of health, quality of life or wellbeing. However, for individuals to provide information on their capabilities requires a judgement to be made by the individuals, *ex ante*, about their ability to do something, rather than *ex post*, about what they currently do. This creates extra complexity in the process of self-reporting the information. Although some piloting of capability questions has been undertaken (Anand et al., 2009; Coast, Flynn, et al., 2008; Coast, Smith, et al., 2008; Lorgelly, Lorimer, Fenwick, & Briggs, 2008), investigations of the response process to capability questions and lay interpretations of the capability concept are lacking. Given the recent interest in assessing capability in healthcare, this study set out to investigate these issues and to draw lessons for future capability measurement work.

## Methods

The feasibility of self-reporting capability was examined by studying responses to the ICECAP-A measure (Al-Janabi et al., 2012). The ICECAP-A measure (reported in the Appendix A) is designed so that individuals can self-report their capabilities across five dimensions of life: ‘stability’, ‘attachment’, ’autonomy’, ’achievement’ and ‘enjoyment’. The measure provides a useful tool for the study reported here because it is designed for completion by the general adult population (rather than a specific group). As a comparator, participants also completed a commonly-used self-reported health (functioning) measure – the EQ-5D (Brooks, 1996). Think-aloud interviews (Ericsson & Simon, 1980; Willis, 2005), followed by a semi-structured interview, were used to investigate the completion of the capability and health measures with a sample of the general public. Think-aloud interviews are one method from a wider family of cognitive survey techniques used to study the way in which “audiences understand, mentally process, and respond to the materials we present – with a special interest in breakdowns in this process” (Willis, 2005, p. 3). In think-aloud interviews, participants are asked to verbalise their thoughts while completing a task. The verbal information then provides an insight into the process of completing the task; potentially enabling the identification of the point at which any problems are encountered. Think-aloud (or cognitive) interviews have been used to explore the process of task completion, initially in relatively complex, multi-step tasks, such as playing chess or unscrambling an anagram (Willis, 2005), but increasingly in understanding the completion of survey questionnaires (Collins, 2003; Ryan, Watson, & Entwistle, 2009; Westerman et al., 2008). The study reported here study used concurrent think-aloud interviews, whereby participants are required to verbalise their thoughts during task completion. Concurrent think-aloud interviews have been shown to generate more information and insights into decision-making processes than retrospective methods (Kuusela & Paul, 2000).

### Sampling

To generate an initial pool of potential participants, an invitation and simple screening questionnaire were sent to 600 randomly-selected individuals from four geographical wards in the UK in 2010. The wards were chosen for maximum socio-economic diversity. The individuals who responded to the invitation (and provided consent) were boosted in the pool by 24 individuals (from the same wards) who had been identified as potential participants in earlier research. No reminders were sent as the initial objective was simply to identify a pool of interested respondents. The resulting pool was purposively sampled on the basis of their responses to the screening questionnaire to ensure diversity in terms of age, sex, ethnicity, health and socio-economic status in the final interview sample. The study protocol was approved by the University of Birmingham's Life and Health Sciences Ethical Review Committee (ERN_08-93).

### Interview conduct

Interviews were conducted in November and December 2010. All but one participant was interviewed in their own home. Participants performed two simple ‘warm up’ think-aloud tasks to familiarise themselves with the technique. In the first task they were asked to count the number of windows in their home (Willis, 2005), thinking out loud as they went. In the second task, participants completed a single five-point question on their general health or life satisfaction. Each participant then received both the ICECAP-A measure and the EQ-5D measure and was asked to complete them thinking out loud as they went (with the order of the questionnaires alternating between participants). Both measures contain five domains referring to an individual's capabilities (in the case of ICECAP-A) and health status (in the case of the EQ-5D). To prompt participants to think aloud, a standard protocol, based on one used by Gilhooly and Green (1996), was then read out, asking participants to verbalise their thoughts during completion of the measures. Participants were not interrupted, but if they were silent for a period of 10 s or more (which was very rare), they were asked to keep thinking aloud. Digital recording was supplemented by written notes on any problems participants encountered or raised while completing the measures.

Following the think-aloud interviews, a semi-structured interview was conducted with each participant. The follow-up interview began with questions to explore the thoughts participants had expressed whilst completing the measures. For example, if participants had queried the relevance of a question, or appeared to struggle to answer it, this issue was explored in the semi-structured interview. After this initial exploration, a topic guide formed the basis for asking further questions to assess whether participants found any aspects of the questionnaire challenging and if so, why. All interviews were transcribed verbatim.

### Interview analysis

The think-aloud portion of each interview was divided into 11 segments; 5 representing the items on the ICECAP-A measure and 6 representing the items on the EQ-5D (including the visual analogue scale question). Four raters then coded the transcripts with the aim of identifying segments of the interview where the participant encountered a problem in the process of completing each question. The survey response model proposed by Tourangeau, Rips, and Rasinski (2000) was used as a coding framework for errors in the task. Tourangeau et al. (2000) propose that in appropriately answering a question, an individual must: (i) understand (comprehend) the question in the way that the researcher intended; (ii) successfully retrieve the appropriate information to answer the question from their long-term memory; (iii) correctly judge how the recalled information should be used to answer the question; and (iv) format the information into a valid response for the questionnaire. Each rater then coded the segments in each transcript as either: (a) error-free, (b) containing one or more errors or (c) as a ‘struggle’. The struggle category was used to identify segments where the participant clearly had difficulty answering the question, but eventually reached an appropriate answer. Consistency between raters on the coding of the data was then assessed using raw agreement and kappa statistics (Cohen, 1960). Following the independent coding, segments were judged as errors (or struggles) if a majority of coders noted a specific type of error (or struggle). Segments where raters were evenly divided, or where there was no majority on the type of error, were discussed and a code agreed upon by all raters.

The qualitative data from the subsequent follow-up interviews were coded and analysed using constant comparison to derive explanatory themes (Strauss & Corbin, 1990). To develop codes, two members of the study team independently coded a subset of transcripts to identify recurrent and salient themes. A coding framework was then agreed on, and applied to the full set of interviews. Additional codes were identified where the data were not well-captured by the initial framework. The coding exercise allowed the data to be categorised into related groups. This facilitated the comparison of related passages of the interview across participants. An account containing both descriptive and explanatory narrative (Ritchie & Lewis, 2003) was developed, to categorise and analyse quotes. Quotes were selected to illustrate the themes and any key differences in the accounts provided by participants in the interviews. Data management was undertaken using Atlas.ti qualitative data management software, to facilitate the coding of interviews and retrieval of coded segments for analysis.

## Results

The invitations to participate generated 51 responses, resulting in a total sample of 75 individuals, when supplemented with the responses from a previous study. A disproportionate number of responses came from respondents in more socio-economically advantaged wards, from women, and from individuals aged 45 and over. From the full sample, 34 individuals were selected to generate a diverse group of participants. Interviews were completed in November and December 2010 and lasted between 15 and 60 min. The characteristics of the interview sample are shown in Table 1.

Following independent coding of the think-aloud interviews by four raters, inter-rater agreement was similar for both the ICECAP-A (79–91%) and EQ-5D (81–92%), with the chance-corrected agreement being rated ‘fair’ to ‘moderate’ using standard guidelines (Landis & Koch, 1977).

### Description of errors and struggles

Table 2 summarises the errors and struggles on the ICECAP-A. With 34 participants each completing 5 questions on the ICECAP-A, 170 segments in total were generated (with 204 segments generated by the 34 participants completing 6 questions on the EQ-5D). Six (3.5%) out of the 170 segments of the ICECAP-A were associated with an error and 10 (5.9%) with a struggle. Of the errors, 3 were response errors, 2 were comprehension errors and 1, a judgement error. Table 3 shows the final error and struggle pattern on the EQ-5D. Twelve (5.9%) out of the 204 segments of the EQ-5D were associated with an error and 3 (1.5%) with struggle. Of the errors, 11 were response errors and 1 was a comprehension error. No retrieval errors were identified on either questionnaire, as expected given the recent recall period. In most cases items were completed, across both the ICECAP-A and EQ-5D questionnaires, without error or struggle. Comments about the questions such as the ones below (which each refer to both questionnaires) were fairly typical:

*I think they're pretty straightforward…* [TA01]

*I think they were all what you might expect from this sort of questionnaire*… *I don't think anything was difficult.* [TA02]

*Oh they were fine, very straightforward and easy to understand.* [TA08]

Tables 2 and 3 suggest that certain types of error or struggle were more likely. For ICECAP-A, struggles on the “attachment”, “autonomy” and “achievement” questions account for just over half of all problems. For “attachment” and “autonomy”, participants struggled to identify the meaning of some of the terms used:

*… I can have a lot of love, friendship and support, I can have quite a lot …I don't really understand what the word can is doing in this question.* [TA37]

*Again ‘independent’ meaning*…*meaning what? Financially, mentally? Physically? If it's mental or physical I'm totally independent, if it's financially I am married so you're never completely independent.* [TA67]

On ‘achievement’, some participants questioned the relevance of the terms at their stage of life:

*I can achieve and progress – at my age there's not much chance of taking degrees, achieving things and progressing forward. You've done all that in years gone by. It's awkward to answer for a pensioner.* [TA41]

In the EQ-5D, fewer problems were encountered in establishing the meaning of terms, but more problems were encountered in selecting an appropriate response category. Response errors accounted for the majority of all problems recorded on the EQ-5D and were generally encountered because the response options failed to fit the experience of the participant:

*… it's actually quite a difficult thing to say because sometimes it depends, my knees are a bit tricky in the winter, but I wouldn't say I have extreme pain or discomfort. But if it said occasional or on a scale of or how often, I don't know … If it said chronic pain then no. If it says moderate pain occasionally then maybe…* [TA02]

*Okay, well I'm thinking of breaking it [the rating scale question] up again as I did in the first [practice] question, and I'm going to write underneath ‘general’ and ‘mental’, from there I'll do the arrows as stated to the points that I think suit*… [TA43]

### Thematic analysis of completion

This section draws on the thematic analysis of both the think-aloud and semi-structured elements of the interviews. Themes were drawn out from the data to explain participants' responses to the two questionnaires. Key among these, and central to the interest of this paper, were themes relating to the response to questions phrased in terms of capability. Other themes, notably ‘question specificity’ and ‘adaptation’ emerged in the thematic analysis and these were also relevant factors for how capability questions, in particular, were answered.

### Capability concept

A number of participants demonstrated, in thinking aloud, that they clearly understood that the capability questions were asking about what they *could* do (or have), rather than necessarily what they *did* (or had). There were examples relating to most questions, of participants noting that they were potentially capable of functioning at a higher level than they did:

*I would be able to be independent in many things but I am probably only independent in a few things. That is most probably my choice…* [TA46]

*I can have a lot of love, friendship and support. That doesn't mean necessarily… I do have it.* [TA03]

*I can achieve things, I can achieve, I suppose yes, if I wanted to, I can achieve mainly and progress in many aspects of my life.* [TA49]

*I can have a lot of enjoyment and pleasure. And it depends on how much I make of it myself.* [TA49]

Participants verbalised their understanding of capabilities in different ways. For example, TA67 suggested that the capability questions could be interpreted about their current ‘capacity’ or ‘control’:

*You can have*… *as in your capacity to have?* [TA67]

*Yeah, well that's down to me isn't it? I can achieve and progress in anything I want to… knowing that I can control myself.* [TA68]

Other participants interpreted the questions as asking more about the future in terms of their ‘potential’ or ‘aspirations’:

*Is this your potential to have?* [TA67]

*Achieve and progress in all sectors is what aspirations I have in a way and I'm just trying to think what my aspirations are.* [TA03]

Subsequent discussion of participants' answers also revealed some cases in which the participant's functioning differed from their capability. For example, one participant reported that she had full capability for ‘attachment’ (in the think-aloud portion of the interview), but later revealed how she chose to restrict her functioning:

*I can have a lot of love, friendship and support*…*but I'm afraid I'm a bit picky and choosy. I don't crave people's company all the time.* [TA64]

Similarly, further probing of TA01's responses revealed that, although he was capable of being independent in all things (and answered the question as such), he would hate to *have* to be:

*Oh yes, yeah I can be, definitely. I'd hate to think I'd have to be independent*… *I can cook and things*… *I can do the washing, ironing, stuff like that but I hate to be the cook but I can do it.* [TA01]

Although most participants did not seem to encounter, or identify, any problems with the wording of the ICECAP-A questions, a number were confused by the word ‘can’ in the ‘attachment’ (and to a lesser extent ‘enjoyment’) question. Some participants commented that the use of the word ‘can’ made the question ‘strange’, ‘funny’, ‘odd’ or ‘vague’. One participant, for example, questioned if he was being asked whether he *would like* ‘attachment’,

*I don't understand what it is ‘I can have’. What is that supposed to mean? Because does it mean ‘I can have it, I'd love it please, bring it on’. Or does it mean ‘I can do with a lot of love and support’? What does ‘I can have’ mean?* [TA49]

One participant interpreted the question as asking whether he was worthy of ‘attachment’ and then answered the question in terms of how much ‘attachment’ he did in fact have:

*I don't really understand what the word can is doing in this question, I don't know whether it means that I am worthy of having a lot of love, friendship and support or what but assuming that it means that I do have a lot of love, friendship and support, I'd say that I have quite a lot of love, friendship and support.* [TA37]

### Question specificity

Both questionnaires aim to cover broad and complex concepts in a small number of simple questions. Some participants picked up on this issue, finding the concepts embodied in the capability questions less specific than they would have liked:

*I think there were lots of areas where I want to say, ‘define things’… all of those questions I felt were very kind of nebulous and not as specific as I would have perhaps liked. Because the more specific it is, the easier it is to answer.* [TA10]

Although some comments were made about the subjectivity of the EQ-5D questions, there was a perception among many participants that the capability questions were the more subjective:

*If you're feeling settled and secure, it's very subjective*… *whereas with the mobility either you can walk about or you have problems walking about or you can't walk about at all, so that is more factual.* [TA37]

While the broad, subjective terms in the capability questionnaire caused some participants to struggle, they did enable participants to bring a large range of factors, which influenced their wellbeing into their answers:

*Right, achievements and progress… it can be like achievement and progress within your work remit or it can be achievement and progress within your own personal life or a combination of the two I suppose.* [TA54]

*I enjoy going out for the day or still going for a meal or going out for lunch, travelling, just seeing the world, new experiences*… *yeah I think I have a lot of enjoyment and pleasure.* [TA26]

Conversely, because the health questions were perceived as more specific, participants were more readily able to retrieve their specific health experience. As a result, they were more likely to identify potential inconsistencies between their experience and the categories on the EQ-5D.

### Adaptation

A number of participants showed signs that they had adapted to their situations. Although longitudinal data were not collected, some participants indicated that the way they would conceptualise the question would differ depending on their circumstances. For the capability questions, this primarily related to participants reinterpreting the ‘achievement’ concept as they got older:

*I mean when I was young I was looking forward to sort of having a good job and getting married and having children and things like that. But you know I've done all that now… the only thing [now] is, as I said, is the health thing, being able to achieve that.* [TA08]

*Dad would say something about with his business and work and things whereas*… *to me achievement and progress relate to university stuff and education because I'm just entering the next phase of life, if you know what I mean.* [TA31]

As noted earlier, there were older participants who struggled to see the relevance of ‘achievement’ in their life. It might be that these participants failed to reinterpret the concept sufficiently for their own life. For the health measure some evidence of adaptation was also found. This was most apparent for the mobility question, where participants were clearly using people of their own age as the reference group:

*Well I think you have to relate it [mobility] to the people of your age too, because it's no good comparing yourself with a 20 year old… so, you know I think you've got to compare it with your peer group and I think compared to my peer group I'm probably about average as it were.* [TA03]

*I'm quite mobile actually for me age, although whereas years ago I could go on all day, now I say every half hour I've got to sit down for five minutes, it does affect you that way, you think you can do it, but when you're called to do it you can't do it. Really I've got no problems, there's some people, I'm 77… I've got no complaints whatsoever.* [TA01, selecting ‘level 1’ mobility]

## Discussion

As self-reported capability data start to become available and are used to evaluate wellbeing in the health field, it is important to investigate whether the data are generated in a meaningful way. This study found that individuals largely responded to capability questions in the intended manner and encountered problems (of any sort) on fewer than 10% of the items (similar to the EQ-5D). Some participants had clear difficulties with the capability terminology and this caused them to struggle. However, there were fewer outright ‘errors’ observed in response to the capability questions relative to the EQ-5D, where a number of individuals found that response categories on the questionnaire did not meet with their experiences. It is important to acknowledge that the ICECAP-A measure focuses on a particular set of capabilities and that other measures focus on different sets of capabilities, that are worded in different ways. Nevertheless there are a number of general implications from the study reported here for researchers interested in capability measurement.

The study reported here showed examples of individuals thinking about their capability and in some cases identifying how their capability diverged from their level of functioning. Divergence between capability and functioning occurred, for example, because of apathy (in the case of ‘achievement’), wanting to ‘pick and choose’ when to socialise (in the case of ‘attachment’) and choosing to enter into family commitments (in the case of ‘autonomy’). These examples provide illustrations of the value the capability approach can bring, in taking into account individuals' intrinsic freedom to choose the life they want. The latter case (family commitments) is perhaps more ambiguous as to whether or not the responding individual has real capability in that domain of life. For example, a number of individuals cited family and relationships as *constraints* on their capability to be fully independent. Thus participants differed in terms of the degree to which they perceived certain factors (including their personality and current circumstances) as constraints on their capability. A useful distinction is drawn by Nussbaum between internal capability and combined capability. Internal capability refers to “…so far as the person herself is concerned, sufficient conditions for the exercise of the requisite functions” (Nussbaum, 2000, p. 84). Combined capability on the other hand represents this internal capability combined with the “external conditions for the exercise of the function” (Nussbaum, 2000, p. 85). While the individual who had family commitments had the internal capability for independence, because of the external constraints of having a family, arguably they did not have the combined capability for full independence. An important issue in developing future capability instruments may be to state whether the aim is to measure internal or combined capabilities.

Where participants questioned the capability wording, there were some differences in their interpretation of what capability meant to them. Some talked about their ‘potential’ or ‘aspiration’ and others about their ‘capacity’ or what was under their ‘control’. Although potential seems to imply something that is attainable and aspiration implies something less probable, both words suggest something that could happen at some time in the future. Individuals who generally think in terms of the longer timeframe (potential, aspirations) would be expected to report higher capabilities, all else being equal as a longer timeframe logically generates more capability as well, potentially, as scope to remove any external constraints to attaining the capability. This may not be desirable given the focus in the capabilities literature on real, or actual, abilities in life (Nussbaum, 2000; Sen, 2009). There is an attempt in the ICECAP-A questionnaire to direct individuals to think more in terms of their current capabilities by asking them to focus on their quality of life ‘at the moment’. The findings of this study suggest that (more) explicit direction on timeframes may be useful on the ICECAP questionnaires and others.

In addition to the different interpretations of the constraints and timeframes that were relevant in responding to capability questions, a third challenge is the language used to convey the capability concept. Clear responses, in terms of capability were not observed across the sample: many participants read the questions in terms of their functioning and/or articulated difficulties with the capability concept, most notability on ‘attachment’. It appears that some individuals struggle with the term ‘I can’ as indicating capability. Although the primary dictionary definition of ‘can’ is synonymous with the idea of being able to do something (Chambers, 2008), the word did seem to result in some undesired interpretations, such as ‘can’ signalling permission or worthiness in the case of the ‘attachment’ question. This suggests careful attention needs to be paid to the way in which capability questions are communicated, with further exploration of the most appropriate terminology. As capability questionnaires are beginning to be translated (Anand, Krishnakumar, & Bich Tran, 2011; Makai, Brouwer, Koopmanschap, & Nieboer, 2012) there is a continued need for qualitative, exploratory work to convey a consistent understanding of the capability concept.

Problems were noted not just in comprehending the capability terminology, but also in understanding some of the terms such as ‘independent’, ‘secure’, and ‘achieve’. While total misunderstandings were very rare, these general terms often prompted participants to speculate on what these meant in the context of the questionnaire. The *literal* meaning seemed to be understood, but there was sometimes doubt about the *intended* meaning, and this manifested itself in some divergent interpretations of capability questions and ‘struggles’. Concern over the potential subjectivity of certain questions has prompted some researchers to switch from asking questions about capabilities in broad domains of life to more specific questions (Anand et al., 2009; Anand & van Hees, 2006). While specific indicators might be appropriate in some contexts, in others contexts, such as developing outcome measures for economic evaluation, it is necessary to try to capture a very broad concept e.g. wellbeing or health, in a small number of dimensions (Brazier, Ratcliffe, Salomon, & Tsuchiya, 2007). Questions about broad concepts, some of which participants clearly did not discuss on a regular basis, also appeared to lead to more thoughtful answers (as judged by the length of the segments on the capability measure). This may be considered an advantage of asking more general questions. Indeed some participants appeared to engage in a process of constructing their capability responses using the ICECAP-A questionnaire as an aide. This contrasted with the health questionnaire. With the EQ-5D, participants were more likely to respond to the questionnaire with clear ideas about their own situation and therefore to identify inconsistencies between their situation and the available options on the questionnaire. This could explain the greater occurrence of response errors on the EQ-5D compared to the capability questionnaire.

In addition to concerns about the understanding of the questions, there were also concerns about the degree to which individuals' adaptation may affect their responses. In this study, two types of adaptation were apparent. On the capability measure, participants sometimes reinterpreted the meaning of terms depending on the stage of life they were at. Most notably, older respondents tended to reinterpret the meaning of ‘achievement’ in their life, focusing on achievements in health, hobbies and family as they aged, rather than paid work. However, this adaptation did not lead to lower standards for judging their capability to achieve, or indeed capability in other domains of life. On the other hand, on the EQ-5D there was more evidence of a shifting reference point, with participants using their own age group or expected abilities as a yardstick for evaluation. This is consistent with previous evidence that has found that people identify poorer mobility, for example, as more acceptable as they age (Brouwer, Van Exel, & Stolk, 2005). Both forms of adaptation can pose measurement problems, but the reinterpretation form, ‘gamma’ change (Allison, Locker, & Feine, 1997; Golembiewski, Billingsley, & Yeager, 1976) poses less of a problem because it is hard to claim that the conceptual reinterpretation is any less valid (or likely to alter scale scores in a systematic way). However, the latter adaptation, caused by a shifting reference point (termed ‘beta’ change), poses more of a problem because external standards are being lowered or shifted in a systematic way, and this may lead to under-reporting of quality of life problems as one ages or get unhealthier. It therefore seems that, although both self-reported capabilities and self-reported health can be subject to adaptation, it is the expectation-lowering adaptation that occurred in the health measure that is more concerning from a normative perspective (Menzel, Dolan, Richardson, & Olsen, 2002; Sen, 2002). Nevertheless, there is ongoing debate in normative economics and health circles about the degree to which adaptation poses problems for using individuals' judgements about their wellbeing for societal decision-making (Hausman, 2010; Kahneman & Sugden, 2005).

The reported study presents an initial investigation into whether capabilities can be self-reported, and a number of issues warrant further reflection. First, one may be concerned that the think-aloud technique encouraged individuals to express thoughts about the measures that they would not have had otherwise. While this cannot be discounted, verbalisation is thought to affect cognitive processes “only if the instructions require verbalisation of information that would not otherwise be attended to” (Ericsson & Simon, 1980, p. 215). Second, this sample contained relatively few individuals in poor health states. For a more thorough examination of capability response in a healthcare setting, one may wish to replicate the study with more individuals in poor health or examine the response to the questionnaire in a particular clinical group. It may be that individuals with severe impairments would react differently to the wording, or responses would differ depending on the context in which the questions were asked. Finally, it must be noted that for expediency, this study focused on capabilities that were self-reported using a specific tool (the ICECAP-A measure). While many of the issues identified here are likely to be relevant more generally, researchers measuring capabilities in different ways (i.e. with other measures or in an interview, rather than paper-based manner) may identify new issues. For example, the questions in the ICECAP-A focus on somewhat abstract issues (achievement, independence etc.). One may be even more optimistic about the understanding of respondents in relation to more concrete capability questions (e.g., ‘are you able to walk to the shops?’).

In summary, this study illustrates that individuals can understand and respond to questions about their capabilities. They can identify where their capability and functioning may diverge (for example in relation to their autonomy) and translate the capability concept into a lay understanding. However, some individuals struggle with certain aspects of self-reporting capabilities, and provide different interpretations of the questions. These difficulties are not confined to questions about capabilities, as this study also demonstrated that problems were similarly encountered on a standard health questionnaire. Nevertheless, greater guidance in the interpretation of capability questions may be important when using capabilities to evaluate outcomes and wellbeing in the health sphere, where issues of bias and comparability are paramount. In general, this study suggests self-reporting of capabilities is feasible and meaningful, but that attention needs to be paid to the wording used to evoke capability and on guiding individuals on the timeframe and constraints they ought to consider in evaluating their capabilities.

## Figures and Tables

**Table 1:** Characteristics of the interview sample.

Characteristic	Participants (*n* = 34)
Sex	
Male	16
Female	18

Native English speaker	
Yes	29
No	5

Age group	
>65	11
45–64	15
<45	8

Local area deprivation (quartile)	
Top (most affluent areas)	10
Second	8
Third	9
Bottom (most deprived areas)	7

Health status on EuroQoL-VAS	
>90	9
71–90	12
51–70	7
50 or less	4

**Table 2:** Errors and struggles on the ICECAP-A capability measure.

	Stability	Attachment	Autonomy	Achievement	Enjoyment	Total
Errors						
Comprehension	0	1	0	1	0	2
Retrieval	0	0	0	0	0	0
Judgement	0	1	0	0	0	1
Response	1	0	2	0	0	3

Struggles	1	3	3	3	0	10
Total	2	5	5	4	0	16

**Table 3:** Errors and struggles on the EQ-5D health questionnaire.

	Mobility	Self-care	Usual activities	Pain	Anxiety	VAS	Total
Error							
Comprehension	0	0	1	0	0	0	1
Retrieval	0	0	0	0	0	0	0
Judgement	0	0	0	0	0	0	0
Response	3	1	0	3	1	3	11

Struggle	0	0	0	1	2	0	3
Total	3	1	1	4	3	3	15
