# Supplementary material for: Can capabilities be self-reported? A think aloud study
Source: Soc Sci Med. 2013 Jun;87:116–22. doi: 10.1016/j.socscimed.2013.03.035 (PMC3664929; doi:10.1016/j.socscimed.2013.03.035)
Supplement: Supplementary file 1 [file mmc1.docx]

**Appendix A: The ICECAP-A questionnaire**

**ABOUT YOUR OVERALL QUALITY OF LIFE**

Please indicate which statements best describe your overall quality of life at the moment by placing a tick (✓) in **ONE** box for each of the five groups below.

| **1. Feeling settled and secure** |  |  |  |
| --- | --- | --- | --- |
| I am able to feel settled and secure in **all** areas of my life |  |  | ^4^ |
| I am able to feel settled and secure in **many** areas of my life |  |  | ^3^ |
| I am able to feel settled and secure in **a few** areas of my life |  |  | ^2^ |
| I am **unable** to feel settled and secure in **any** areas of my life |  |  | ^1^ |
|  |  |  |  |
|  |  |  |  |
| **2. Love, friendship and support** |  |  |  |
| I can have **a lot** of love, friendship and support |  |  | ^4^ |
| I can have **quite a lot** of love, friendship and support |  |  | ^3^ |
| I can have **a little** love, friendship and support |  |  | ^2^ |
| I **cannot** have **any** love, friendship and support |  |  | ^1^ |
|  |  |  |  |
|  |  |  |  |
| **3. Being independent** |  |  |  |
| I am able to be **completely** independent |  |  | ^4^ |
| I am able to be independent in **many** things |  |  | ^3^ |
| I am able to be independent in **a few** things |  |  | ^2^ |
| I am **unable** to be at all independent |  |  | ^1^ |
|  |  |  |  |
|  |  |  |  |
| **4. Achievement and progress** |  |  |  |
| I can achieve and progress in **all** aspects of my life |  |  | ^4^ |
| I can achieve and progress in **many** aspects of my life |  |  | ^3^ |
| I can achieve and progress in **a few** aspects of my life |  |  | ^2^ |
| I **cannot** achieve and progress in **any** aspects of my life |  |  | ^1^ |
|  |  |  |  |
|  |  |  |  |
| **5. Enjoyment and pleasure** |  |  |  |
| I can have **a lot** of enjoyment and pleasure |  |  | ^4^ |
| I can have **quite a lot** of enjoyment and pleasure |  |  | ^3^ |
| I can have **a little** enjoyment and pleasure |  |  | ^2^ |
| I **cannot** have **any** enjoyment and pleasure |  |  | ^1^ |
|  |  |  |  |

Please ensure you have only ticked **ONE** box for each of the five groups.

© 2010 Hareth Al-Janabi and Joanna Coast
